# Internal versus external determinants of *Schistosoma japonicum* transmission in irrigated agricultural villages

**DOI:** 10.1098/rsif.2011.0285

**Published:** 2011-07-13

**Authors:** Robert C. Spear

**Affiliations:** Center for Occupational and Environmental Health, School of Public Health, University of California, Berkeley, CA 94720-7360, USA

**Keywords:** *Schistosoma japonicum*, environmental factors, mathematical model

## Abstract

Currently schistosomiasis transmission has been suppressed to low levels in many historically endemic areas of China by widespread use of praziquantel in human and bovine populations and application of niclosamide for snail control. However, re-emergent transmission has signalled the need for sustainable interventions beyond these repeated chemical interventions. To take advantage of ongoing investment in rural infrastructure, an index of schistosomiasis transmission potential is needed to identify villages where environmental modifications would be particularly effective. Based on a retrospective analysis of data from 10 villages in Sichuan Province, an index linked to the basic reproductive number is shown to have promise in meeting this need. However, a lack of methods for estimating the spatial components of the proposed metric and for estimating the import of cercariae and miracidia from neighbouring villages leads to significant uncertainty in its estimation. These findings suggest a priority effort to develop methods for measuring the free-swimming forms of the parasite in surface waters. This need is underscored by the high cost and limited sensitivity of current methods for diagnosing human infection and mounting evidence of the inadequacy of snail surveys to identify environments supporting low levels of transmission.

## 1. Introduction

Rural areas of China have recently benefited from increased governmental expenditures in disease control programmes, particularly in the wake of the severe acute respiratory syndrome (SARS) experience. These benefits have extended to the control of human infections owing to *Schistosoma japonicum*, where expanded programmes have continued to rely on the treatment of human and bovine populations with praziquantel and the use of the molluscicide niclosamide, but with substantially intensified use of both. Overall, these efforts have suppressed transmission intensity, often below the detection levels of routine surveillance [1]. However, re-emergent transmission in areas formerly meeting the Chinese government's criteria for transmission control or interruption has signalled the need for a more comprehensive approach, which includes environmental changes of the sort already taking place, often for unrelated reasons [2–4]. In order to influence rural development priorities for disease control purposes at least a semi-quantitative understanding of the effects of environmental modification on disease transmission is needed. The objective here is to address this practical need by synthesizing over 15 years of collaborative research on the factors controlling the intensity of transmission of schistosomiasis in irrigated agricultural environments of southwestern China.

Previously, our group defined three principal determinants of the intensity of the disease transmission cycle within a village. They were termed *internal potential, gating effects* and *connectivity* [5]. The first two of these determine the ability of a village isolated from external parasite inputs to sustain transmission between resident humans and snails. Connectivity acknowledges that villages seldom exist in isolation, but are connected at some level to their neighbours and the broader environment by a variety of pathways, notably hydrological connections and human and animal pathways that can transport the parasite into and out of the village. Indeed, a fundamental issue addressed here is whether these connections are sufficiently strong to demand that schistosomiasis transmission, and hence its control, must be viewed in this environment from a metapopulation perspective [6,7]. In that context, the populations are those of parasites which colonize hosts, both molluscan and mammalian, who constitute patches that are distributed geographically in villages. Viewed from either the metapopulation or the disease control perspective, a central question is one of scale. In a village, to what extent is transmission driven by internal versus external factors? If transmission is dominated by external factors, attention must be focused at a scale larger than that of a single village, one which includes the sources of these imported parasites and the mechanisms by which they are dispersed. An early example from among the villages analysed here was the very rapid and extensive re-infection observed over a 1 year period following thorough and comprehensive praziquantel treatment of all residents. This occurred in a village with major irrigation water input from an upstream village in a different county in which no interventions were known to have been conducted in the recent past. External inputs were thought to be responsible for the rapid re-infection and these were expected to maintain transmission at high levels, with or without continued internal control activities, unless the upstream village(s) was also included in the control programme. A goal of the study reported here was to explore the validity of that interpretation of events in a focused analysis and on a broader scale.

Through the lens of the foregoing concepts, an early but comprehensive dataset on disease transmission in 10 villages in the years 2000–2002 is revisited. The first objective is to assess the adequacy of field data typically collected in endemic rural villages in identifying the internal determinants transmission intensity. The second objective is to explore the necessity of the metapopulation perspective in understanding the distribution of endemic disease. There is little doubt that a metapopulation approach is necessary to understand the dynamics of re-emergent transmission. Anticipating that the field data analysed here, which are at the upper limit of what is routinely available, will be found inadequate to resolve either issue definitively, a key question is how these inadequacies can be addressed through the development and deployment of improved methods to inform the final effort to achieve sustainable interruption of schistosomiasis transmission in China.

### 1.1. Epidemiological setting

The villages discussed here are located in Xichang County in southwestern Sichuan Province. They are irrigated agricultural villages typical of the hilly and mountainous classification of schistosomiasis transmission ecologies used in China. The amphibious snail *Oncomelania hupensis robersoni* is the intermediate host of the parasite and lives mainly in irrigation ditches. Exposure to humans occurs in either agricultural or domestic activities involving contact with ditch water. Conversely, snails are exposed, also mainly in the ditch environment, to miracidia which hatch from schistosome eggs, many of which are distributed into the village environment via the use of human and animal waste for crop fertilization. In earlier studies, we found that the determinants of human infection were principally related to the agricultural characteristics of the village of residence and, secondarily, to one's occupation within the village. Neither age, gender, or kinship appeared to be significant determinants of individual risk within a village nor did the small resident domestic animal populations appear to be important in sustaining endemic levels [8,9]. Many of the foregoing findings were made possible because of the very limited control activity in these villages in at least the 5 years prior to the initial surveys in 2000. Hence, the situation as found in that year was assumed to represent steady-state or near steady-state transmission, which is also assumed in the analyses described here.

Fundamental to the analysis presented below is that these villages were originally selected because they were known to present a range of endemic disease transmission from low to high intensity. The initial studies were concerned with identifying the internal determinants of these different levels of disease transmission. Hence, spatially contiguous villages were excluded because their parasite-related connections with their immediate neighbours were not the focus of interest at the time. For present purposes, however, a qualitative picture of the setting of these villages can be gained from the map included in the electronic supplementary material while noting that the farmland surrounding virtually all of these villages is immediately adjacent to that of other villages not shown.

## 2. Methods

A differential equation model of the type extended and popularized by Anderson & May [10], but incorporating pathogen import/export terms, was used both to develop an index of internal transmission intensity and to explore the balance of internal versus external determinants of the endemic level of disease in these villages. The modelling approach taken here acknowledges the network structure implied by the import and export of larvae between communities while, as will be seen, remaining compatible with the available data on such exchanges. While current research has begun to address patterns of movement of humans and bovines [11] as well as hydrological transport of the parasite between villages, the focus here is on quantitative evidence of the relative importance of these connections to sustaining endemic disease transmission.

The version of the model used here, equations (2.1), is parametrically complex because it was developed to incorporate site-specific descriptors of the infection cycle. It also includes acquired immunity as well as the aforementioned gating and connectivity effects although the effects of immunity have only modest impacts on the results of this analysis [12]. The state variables are village mean worm burden in humans, *w*(*t*), mean village density of infected snails, *z*(*t*) and the mean level of an index of acquired immunity among the human population, *I*(*t*). Connectivity is represented within the quantities in square brackets in the first two equations, which are the sum of the internally generated and imported cercariae and miracidia, respectively. Both of these free-swimming forms of the parasite are found in the village irrigation system where mammalian and snail exposure takes place.2.1
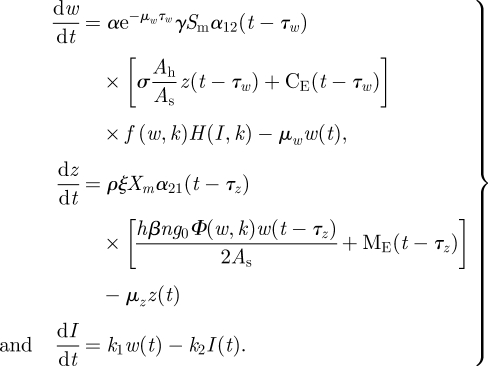


The constant parameters of the model are defined individually in table 1, but there are several phenomenologically important inputs and parameter types:

— external inputs of cercariae and miricidia, C_E_(*t*) and M_E_(*t*), respectively, represent the spatially averaged sum of all sources of each of the two forms of the parasite which may be imported into a village and potentially infect either resident snails or mammalian hosts. Either form may be present in irrigation water entering the village or, for miracidia, faecal inputs from infected bovines or reservoir hosts may be deposited and hatch within the village environment. Assuming no internal losses, the export of cercariae and miracidia from the village at any time *t* is a function of the sum of the two terms in the square brackets of equations (2.1) times the volumetric flow rate of total discharge from the village, although that does not figure into the analysis below;
— time delays, *τ*_*w*_ and *τ*_*z*_; the development time of the parasite to an adult worm in the human host and the environmentally driven development time of the parasite in the snail from infection to cercarial release;
— time-variable or gating parameter aggregations; *α*_12_ (*t* − *τ*_*w*_) and *α*_21_ (*t* − *τ*_*z*_), which include seasonal water contact of humans, temperature-dependent infectivity of cercariae and miracidia and uninfected snail density. Both are scaled to vary between zero and unity and are discussed in detail by Remais *et al*. [13]; and
— site-specific constant parameters, where a site is defined as a natural village, and site-invariant constant parameters which are assumed at least regionally invariant. Each constant parameter is so classified in table 1.

**Table 1.** Parameter definitions, their site-specific versus site-invariant classification, and values used.

parameter	*P*_s_	*P*_b_	value	definition
*α*		X	0.005	schistosomes acquired (per cercaria per m^2^ contact)
*μ*_*w*_		X	0.001	worm natural mortality (per day)
*τ*_*w*_		X	30	development time of worms in human host (day)
*γ*	X		see text	spatial index for the distribution and interaction between exposure and cercariae
*S*_m_	X		table 2	annual maximum water contact index
*σ*		X	35	cercarial production (per sporocyst per day)
*A*_h_	X		table 2	snail habitat (m^2^)
*A*_s_	X		table 2	water surface area (m^2^)
*ρ*		X	3 × 10^−6^	intermediate host infection (per miracidium per square metre surface water)
*ξ*	X		see text	spatial index for the distribution and interaction between snails and miracidia
*X*_m_	X		table 2	annual maximum uninfected snail density (snails m^−2^)
*H*		X	1.8	eggs excreted (per worm pair per gram faeces)
*β*	X		0.5–1.0	fraction of human and animal waste recycled
*N*	X		table 2	village population
*g*_0_		X	90	human faecal production (gram per person per day)
*A*_s_	X		table 2	water surface area (m^2^)
*μ*_*z*_		X	0.02	patent and latent snail death rate (per day)
*K*	X		0.45	negative binomial aggregation parameter
*K*_1_		X	0.00142	immunity development rate (immunity per worm per day)
*k*_2_		X	0.0142	immunity decay rate (per day)
*γ*_*w*_		X	0.002	density limitation of worm establishment
*τ*_*z*_	X		variable	parasite development delay in snail (days)
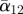	X		0.309	annual average (averaged model)
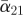	X		0.058	annual average (averaged model)

The nonlinear functions *Φ*(*w,k*), *f*(*w,k*) and *h*(*I*) describe, respectively, worm mating probability, density-dependent worm establishment *in vivo* and acquired immunity. Following Anderson & May [10]:2.2
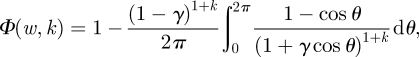
where *γ* = *w*/*w* + *k* and:2.3
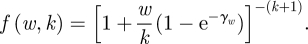


All three nonlinear functions assume a negative binomial distribution of worms in the human population and this is implicit in the foregoing functions. The function *H*(*I,k*) is described in Wang [12] for individuals and a community-level example is shown graphically in the electronic supplementary material under the negative binomial assumption with dispersion parameter *k* = 0.45, which describes a highly right-skewed distribution.

### 2.1. An index of internal transmission

In an earlier analysis of the determinants of the transmission of *S. japonicum*, a distinction was drawn between site-specific and site-invariant factors [14]. Here, the objective is to determine if an index of the village-specific internal transmission potential arising from that distinction is a useful index of the level of endemic steady state within a village, the rate of re-infection after praziquantel treatment, and of the balance between internal and external sources of the parasite. To derive that index, consider an isolated village in which only internal transmission of the parasite occurs. This case is defined for system (2.1) by the condition C_E_(*t*) = M_E_(*t*) = 0. In a stationary environment, the time-variable gating parameters *α*_12_(*t*) and *α*_21_(*t*) are annually periodic. It follows that a non-zero endemic steady-state level of system (2.1) is that in which the state variables are also annually periodic with constant time-weighted average values from year to year. There are two stable steady-state levels of system (2.1), the endemic state and the zero state. For infectious disease transmission systems, the basic reproductive number of the system, *R*_0_, refers to a stability criterion that, for a given parameter set, determines which of these two states the system will approach over time. However, system (2.1) is nonlinear and time-variable with development delays, one of which is itself time-variable being based on a degree-day model of parasite development in the snail. Hence, to derive a necessary and a sufficient criterion for stability of the zero state, a genuine *R*_0_, is a formidable mathematical challenge and beyond the scope of the present study. Given that the objective is to derive an index of the strength of internal transmission, a relative rather than absolute index is sufficient, although a close linkage to the genuine *R*_0_ is desirable. Hence, an approximate index will be derived based on stability criteria for an averaged system in which both the gating parameters and the time-variable external inputs are replaced by their annual time-weighted average values. The averaged system is:2.4
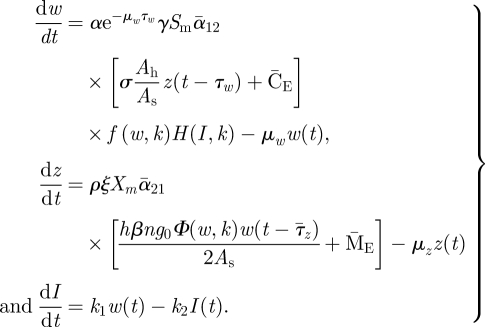


Approximating the steady-state conditions of system (2.1) by equilibrium values of system (2.4) involves products of average values versus average values of time-variable products (see the electronic supplementary material). In distinction to system (2.1), the equilibrium states given by the solutions of the algebraic equations resulting from setting the left-hand side of equations (2.4) to zero can be determined as a function of the constant parameters and stability conditions can be established. Recalling that the operating hypothesis is that the Xichang villages were at or near steady-state in 2000, the accuracy of this approximation, given the parameter values and gating functions for the Xichang region, will be assessed by simulation of equations (2.1) in comparison with the values predicted by equations (2.4) in the context of both the 2000 steady state and the 2002 re-infection studies discussed below. The success, or lack thereof, is intended to point to key data insufficiencies as well as inadequacies in approximating the behaviour of the time-variable system by the averaged model.

For an isolated village where 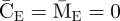, the equilibrium level of human worm burden, 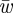, is given by the solutions to a nonlinear algebraic equation derived by first setting the left-hand side of equations (2.4) to zero and using the third equation to express 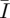 in terms 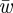, leading to the simplification that 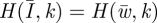. With this substitution, 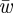 is given by the solutions to the equation:2.5
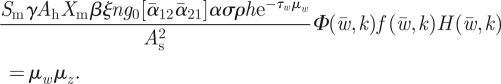


As noted above, the constant parameters occurring in the foregoing equations are divisible into site-invariant and site-specific subsets as specified in table 1. As suggested by equation (2.5), it is convenient to aggregate these subsets into two functional groups. The site-specific group is defined as:2.6
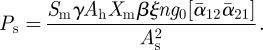


As indicated in table 1, *S*_m_ and *γ* relate to human water contact, *X*_m_ to uninfected snail density, *βng*_o_ to parasite-contaminated fertilizer use and *A*_h_ and *A*_s_ the area of snail habitat and surface water in the village, respectively. Values for *S*,*A*_h_, *A*_s_, *X*_m_ and *n* for the 10 villages studied here are given in table 2 and *g*_0_ is available from the literature [8]. *β* is the fraction of human faeces recycled for fertilization internally in the village. Environmental factors also include temperature and irrigation profiles that alter the long-term average values of the gating parameters *α*_12_(*t*) and *α*_21_(*t*). Generally, irrigation profiles will vary between villages, but are here treated as site-invariant because of a regional pumped irrigation system. In general, however, being site-specific, *P*_s_ is an index which allows for comparison of the intensity of transmission between villages with the same site-invariant parameter subset, *P*_b_. The latter is defined as:2.7
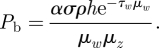

**Table 2.** Village data for 2000 prior to intervention and 2002 re-infection data [8,15].

village	water contact	habitat area (m^2^)	village population	village mean worm burden		uninfected snail density (snails m^−2^)		infected snail density (snails m^−2^)	
				2000	2002	2000	2002	2000	2002
Xinmin 3	2.14	10 350	240	9.7	11.0	11.6	7.4	0.058	0.018
Xinmin 7	1.38	7602	188	103.7	29.6	37.9	15.7	0.227	0.128
Shian 5	2.50	3784	125	90.8	10.7	25	14.0	0.400	0.104
Jianxing 6	1.24	8898	219	59.9	27.6	18.3	14.4	0.378	0.058
Jiaojia 4	1.90	4991	223	4.1	2.4	10.5	4.9	0.105	0
Hexing 1	2.29	10 691	227	25.7	2.0	9.7	14.5	0	0
Minhe 1	2.90	7780	236	16.9	15.1	24.7	9.0	0.049	0
Minhe 3	1.67	7678	194	83.9	23.0	13.5	6.9	0.135	0.030
Xinlong 7	2.67	3878	157	110.4	17.7	20.9	16.4	0.481	0.231
Tuanjie 2	2.14	4278	165	1.1	1.7	1.6	3.8	0	0

The site-invariant parameter estimates listed in table 1, including those pertaining to acquired immunity, are based on calibration calculations reported in earlier work [12,14,16]. Here, a value of *P*_b_ = 2.3 × 10^−2^ is used. It should be noted that the estimates of the spatial parameters derived below are conditioned on the value of *P*_b_ as well as on the degree to which the average value of the gating functions adequately captures their impact on the time-variable system. Hence, while there is uncertainty in the value of this parameter aggregation and the gating parameters, the assumption of invariance across these villages leads to consistent relative rankings based on *P*_s_.

Using the parameter aggregations and defining *Λ* = *P*_s_ *P*_b_, equation (2.4) may be simplified to2.8
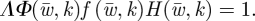


By definition, the nonlinear functions *Φ*, *f* and *H* are all less than or equal to unity, which leads to the conclusion that *Λ* must be greater than 1 for an endemic equilibrium to exist. That property and its specific functional dependence on the system parameters indicate that *Λ* is essentially the basic reproductive number of the averaged system in the absence of the effects of the density-dependent nonlinearities [10]. Note that equation (2.8) can be solved for *Λ* given the values *Φ*, *f* and *H*, which depend only on the equilibrium state, 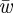 and the village-specific aggregation constant of the negative binomial distribution, *k*. Equations (2.6) and (2.8) produce a second estimate of *Λ* based on the remaining system parameters, which results in two estimates based on different parameter groups. Both will be used below.

**Figure 1.**
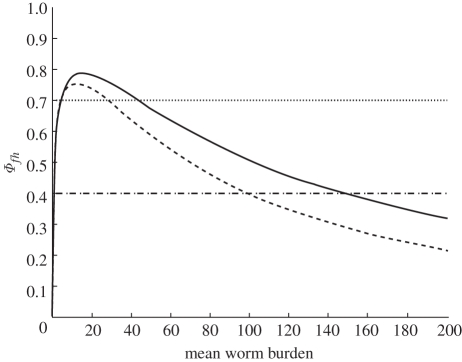
Plots of equation (2.8) for two values of *Λ* and for values of *k* = 0.45 and *γ*_*w*_ = 0.001 and 0.002. Intersection of the *Φfh* curve and the 1/*Λ* lines, together with the (0,0) point, are the equilibrium values of the averaged model, equations (2.4). Solid line, *γ*_*w*_ = 0.001; dashed line, *γ*_*w*_ = 0.002; dotted line, 1/*Λ* = 0.7; dashed-dotted line,1/*Λ* = 0.4.

Turning first to the qualitative aspects of equation (2.8), figure 1 shows the product 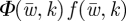 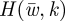 plotted versus the equilibrium values of village mean worm burden 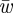 for the parameters used here and thought to be typical for *S. japonicum*. The intersection with the horizontal line 1*/Λ* shows the values of 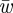 satisfying equation (2.8). Because 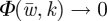 as 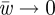, the origin is always a stable equilibrium point. For values of *Λ* less than about 1.3, the infection-free state is the only stable equilibrium point. For values of *Λ* > 1.3, there are two intersections for 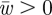, the endemic equilibrium point controlled by the product 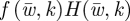 and an intermediate point, sometimes called the breakpoint, which has been shown to be generally unstable [10]. It is of interest from a phenomenological perspective that the endemic equilibrium point can exist for what seem to be quite low values of 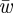 if *Λ* is only slightly in excess of 1.3. This is of some explanatory interest since it can account for the wide range of endemic levels of worm burden observed epidemiologically over quite small geographical areas [8].

### 2.2. Estimating the site-specific parameters

The initial cross-sectional survey data to be used for parameter estimation were obtained in 2000 and covered 20 villages of which the 10 discussed here were followed up in 2002. The year 2000 survey data used here are given in table 2 together with the 2002 re-infection worm burden in humans and results of the 2002 snail survey [13]. The data collection methods and full results are given in Spear *et al*. [8].

While all of the field data are subject to some degree of measurement error, estimates of the infected snail density are based on snail surveys and are particularly problematic because the infection rate in snails, even in highly endemic villages, exceeds 2 per cent only rarely. For example, in only nine of the original 20 villages were any infected snails found in 2000, yet human prevalence in the 11 without infected snails ranged from 3 to 34 per cent. In five of the 10 villages followed up here, between zero and five infected snails were found in samples of between 500 and 1500 total snails. For the villages on the low end of the spectrum, the ratio of the lower to the upper 95% binomial confidence intervals on the infection fraction exceeds an order of magnitude. Hence, in these five villages, the steady-state levels of the infected snail density are poorly estimated and, coupled with the probable levels of within-season variation of infected snail density, the estimates of village mean values are imprecise at best.

Estimates for most of the site-specific parameters are available from the field data (table 2). Further, good estimates of the two time-variable parameters, and hence their average values, exist for the interval 2000–2002 [13]. However, no estimates based on field data are available for the parameters *γ* and *ξ*, which represent exposures that differ from the village mean owing to spatial heterogeneity in the interaction between cercarial density and human water contact (leading to human infection) and between miracidial density and snail contact (leading to snail infection), respectively. In general, neither cercariae nor infected snails are homogeneously distributed across the village irrigation system [8]. Seto *et al*. [15] make a compelling case that *γ* is generally an important descriptor of human exposure. As the only remaining unassigned parameters necessary to estimate the index of internal transmission, *P*_s_, the values of *γ* and *ξ* are important in that they determine the balance between internal and external sources of the parasite. The exceptions are where the infected snail density is zero, in which case only external cercarial inputs are possible, or if a village is classified as isolated, in which case all transmission is internal by assumption. Hexing 1 and Tuanjie 2 are examples of the former, and Shian 5 and Xinlong 7 of the latter. For the remaining six villages, the balance is uncertain. However, for the two isolated villages, it is possible to solve the equilibrium equations of the averaged system for *γ* and *ξ* using the field data in 2000 from table 2 together with the observed values of the equilibrium states 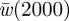 and 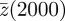. For Shian 5, the mean worm burden was 90.8 with the aggregation parameter of the negative binomial distribution estimated to be *k* = 0.45 [17]. For Xinlong 7, the worm burden was 110.4 also with *k* = 0.45. These values result in estimates of 1.03 and 1.11 for *γ* and 1.83 and 1.89 for *ξ*, respectively.

### 2.3. Estimating external versus internal transmission

For the remaining six villages with infected snails, but which cannot be assumed to be isolated, the data are simply inadequate to estimate these spatial parameters and, therefore, the internal/external balance in any direct way. However, some insight can be gained by determining plausible ranges for *γ* and *ξ*. Again, the equilibrium equations of the averaged system were used, with the constraint that both 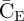 and 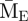 be greater than or equal to zero, to derive upper bounds on both *γ* and *ξ* for each village. These upper bounds correspond to setting both 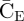 and 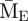 equal to zero and imply that all transmission is internal. Further, the similarity of the estimates of *γ* and *ξ* for the two isolated villages suggests that those values may serve as reasonable central estimates for all villages in the region with similar irrigation systems and agriculture. Proceeding on this assumption, normal distributions were assumed to describe the variability of these parameters among such villages with means set to the average of the two isolated villages, 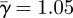 and 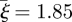, and variances of 0.33 and 0.54, respectively, truncated, if necessary, to insure that the upper bounds were not violated. Given the values of *γ* and *ξ* so generated, estimates of the distribution of *Λ* for each of the six non-isolated villages with infected snails were then calculated from the equilibrium equations.

For any one of the six villages, a random draw from the *γ* and *ξ* distributions allows the calculation of 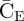, 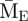 and *Λ* from the equilibrium equations of the averaged model. The final complication is that 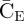 and 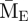 are uniquely determined by the values of *γ* and *ξ*, but *Λ* is a function of their product. Hence, for any values of *γ* and *ξ*, there are many possible values of *Λ* and conversely. While the equilibrium values are invariant at constant *Λ*, simulation experiments using the time-variable system, equations (2.1), showed that the 2 year re-infection rates in human residents of Xinmin 7, for example, varied by 5–10% over the range of values of 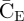 and 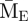 for a given *Λ* (see the electronic supplementary material). The 2 year re-infection rates in snails, in contrast, varied over a twofold range. Hence, the lack of specific knowledge of these external inputs leads to greater uncertainty in the re-infection predictions in contrast to the equilibrium values. In the simulation studies discussed below, the 50th percentile *Λ* from the Monte Carlo runs was used with the *γ* and *ξ* values corresponding to 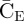 and 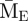 at the approximate median of the range of sampled values for that *Λ*.

To determine the adequacy of approximations based on the averaged system, equations (2.4), the time-variable system including the time-variable developmental delay in snails, *τ*_*z*_, were simulated using Matlab v. 7.7, for eight villages first to assess the match of the equilibrium conditions to those observed in 2000. The two villages with no infected snails in 2000 were not simulated. For the re-infection predictions, the 2002 field data showed that uninfected snail density had declined markedly from the density in 2000. Therefore, for the 2 year re-infection simulations, the uninfected snail density was changed to the average of the 2000 and 2002 snail survey data and simulated over the period from 1 January 2001 to 1 January 2003. For both runs, the initial conditions on worm burden were set to 5 per cent of those in 2000 reflecting an assumed 95 per cent effective rate of praziquantel treatment and 100 per cent coverage of those infected. The same *Λ*, *γ*, *ξ*, 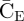 and 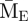 values were used as in the steady-state estimating simulations for each of the eight villages.

## 3. Results

**Figure 2.**
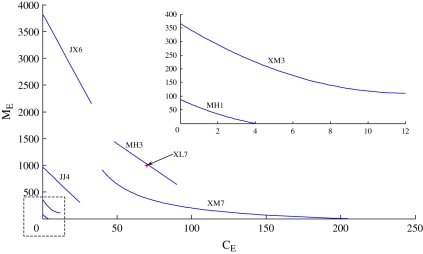
The range of 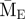 and 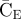 values satisfying the equilibrium equations of the averaged model at the 50th percentile *Λ* for six non-isolated villages with infected snails. The annual average internal values of 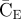 and 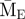 at 2000 equilibrium are shown for the isolated village Xinlong 7 (XL7) as a benchmark. (Online version in colour.)

Table 3 contains the 2.5, 50 and 97.5 quantile estimates from the sample distribution function of *Λ*, using the Quantile routine in R, based on 300 Monte Carlo replications together with mid-range values of 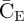 and 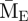 for the six villages where both internal and external parasite sources are possible. To place these numbers in context, figure 2 shows the range of 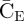 and 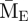 values that are consistent with the sampled values of *γ* and *ξ* at the 50th percentile of *Λ* for each village. As a benchmark, in the 2000 data, model-based estimates of the internally generated cercariae in the highly endemic isolated village Xinlong 7 averaged about 70 cercariae m^−2^ surface water and 1000 miricidia m^−2^ during the transmission season. Jianxing 6 is clearly an outlier in that the miracidial input at the midpoint of the curve in the figure is about three times that of any other village. With that exception, the ranges of the external inputs for the other villages seem plausible in the light of the Xinlong 7 benchmark also shown in the figure. Xinmin 7 (XM7), for example, is the village with the highest external cercarial input and substantial miracidial input. It is also the village mentioned earlier which was thought to have substantial parasite input from an immediately upstream endemic village in Zhaojue County.

**Table 3.** 2.5, 50 and 97.5 percentile ranges of *Λ* and mid-range values of 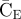 and 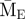 used to generate predictions shown in figure 3*a,b* together with upper confidence limit on probability that village will sustain internal transmission without external inputs.

village	*Λ*	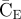	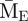	*p*(*Λ* > 1.0)
Xinmin 3	0.050, 0.206, 0.375	2.5	260	<0.01
Xinmin 7	0.108, 0.529, 0.976	80	300	<0.04
Shian 5	2.36	0	0	1
Jianxing 6	0.039,0.188, 0.276	14	3000	<0.01
Jiaojia 4	0.004, 0.058, 0.128	9	630	<0.01
Hexing 1	—	—	—	0
Minhe 1	0.273, 0.966, 1.41	1.6	43	0.62
Minhe 3	0.058, 0.189, 0.284	68	1020	<0.01
Xinlong 7	2.71	0	0	1
Tuanjie 2	—	—	—	0

Also shown in table 3 is the upper binomial confidence limit on the probability that *Λ* > 1.0. This condition is a conservative index of the probability that the village can support endemic transmission without external inputs. Minhe 1 is the only village of the eight non-isolated villages where this analysis suggests that an endemic level supported mainly by internal transmission is a possibility. Solely internal transmission is a possibility given the topographic and hydrologic setting of Minhe 1. However, on the basis of the present analysis, confidence in this result is low because of the low infected snail density. On the other hand, the observed worm burden of 16.9 appears to be on the lower edge of stable endemic levels suggested to be possible by figure 1 and serves to illustrate the low steady-state worm burdens that can apparently occur in this environment. Overall, this analysis suggests that at least seven of these 10 villages received parasite inputs from external sources and, further, none of these appear likely to have sustained internal transmission without these inputs.

The re-infection trajectories of worm burden, *w*(*t*), infected snail density, *z*(*t*) and acquired immunity, *I*(*t*), for the two isolated and six non-isolated villages over 15 years post-treatment are shown for the 50 percentile value of *Λ* in the electronic supplementary material. Since the intent was to determine if the 2000 conditions were consistent with the equilibrium values of the averaged model, it is the steady-state value determined by simulation that is compared with the equilibrium value of the averaged model. Hence, these trajectories assume an unchanged environment based on the 2000 data. This is the case because no system parameters are altered by praziquantel treatment nor was the uninfected snail density assumed to have been changed in these simulations.

**Figure 3.**
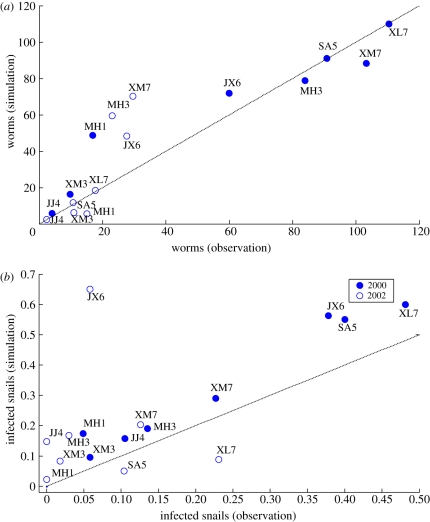
(*a,b*) Year 2000 equilibrium levels and 2002 re-infection levels of worm burden and infected snail density predicted by the averaged model using the 50th percentile *Λ* and mid-range 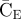 and 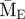 levels from table 3 versus those simulated by the time-variable model. (Online version in colour.)

Figure 3 shows model-predicted versus observed levels of worm burden and infected snail density for both the 2000 steady-state levels and the 2002 re-infection simulations. The former are year-long averages at steady state and the latter the values on day 730. Here, the 2000 levels at the upper end of each panel are the simulated steady-state levels that should correspond to the initial conditions in 2000 prior to treatment. As expected, the 2002 re-infection levels lie nearer to the origin in figure 3. The 1 : 1 line on each panel of figure 3 indicates perfect correspondence of the prediction resulting from simulation of the time-variable model versus the observed data. Because the timing of both human and snail infection surveys occurred over roughly a month in the 10 villages, the specific values of the simulated re-infection levels in 2002 are approximate. This uncertainty is substantial for the infected snail density owing to the large within-year variation seen in the simulations and is reflected in the greater deviation from the 1 : 1 line for this variable shown in figure 3*b*.

The particularly notable features of figure 3*a* are that the isolated villages lie on the 1 : 1 line for both the 2000 and 2002 data. Indeed, the fit is reasonable for all the 2000 data with the exception of Minhe 1. It appears that the low observed endemic level in that village makes it particularly sensitive to the small external input used in this simulation as suggested by further simulations, which showed a very good fit for no external input, but with similar *γ* and *ξ* values. The major discrepancies are in the 2002 re-infection data in the three villages with high external inputs, as shown in figure 2. The three villages, those lying well above the 1 : 1 line in figure 3*a*, indicate that the model produces much higher rates of re-infection than indicated by the field data. This can be seen by comparing the re-infection rates in the village-specific simulations of the isolated Shian 5 versus that of Xinmin 7, shown in the electronic supplementary material. The time to near equilibrium in Shian 5 is about 12 years and in Xinmin 7 about 4 years, the latter having high external inputs. The higher predicted re-infection levels may also be due to the fact that the external inputs are treated as constants in both versions of the model whereas that is unlikely to be the case in the field. Whatever the reason, the clear message is that field data are needed on the external inputs, and *γ* and *ξ* in order to further explore the adequacy of the modelling approach or the other data on which parameter estimates are based.

In contrast to the worm burden predictions in figure 3*a*, figure 3*b* shows that the snail density in the isolated villages is overestimated in 2000 and underestimated in 2002 by the *γ* and *ξ* parameters produced by the averaged model. Jianxing 6 is clearly an outlier as suggested by its external input profile in figure 2. However, all other villages show higher predicted than observed snail densities consistent with recent field data, collected using the same snail sampling protocols, which showed no infected snails in 35 of 36 re-emerging villages with human infections [1]. The model results, the original field data, and the more recent field data all indicate that these standard protocols have a limit of detection of infection prevalence in snails above that where continued human transmission occurs.

## 4. Discussion

Recalling that the results shown in figure 3 were obtained without curve fitting or village-by-village optimization, the use of the average model and its site-specific parameter set, *P*_s_, provide a reasonable index of the environmental capacity of a village and its residents to support transmission of schistosomiasis when adjusted for external inputs and with at least approximate estimates of internal spatial parameters. This index is simple and its control implications are easily understood. However, with the current data limitations, it leads to marginally useful short-term re-infection estimates for villages suspected of having substantial external inputs. All of these results were obtained based on the assumption of steady-state transmission in these villages in 2000 as well as the fortuitous inclusion of two isolated villages in the dataset. Given the widespread recent use of praziquantel in China, steady-state data are unlikely to be available in other settings for the foreseeable future. Hence, direct data from the field on C_E_(*t*), M_E_(*t*) and the two spatial parameters will generally be needed to achieve quantitative assessments of local transmission intensity. Moreover, the foregoing results suggest that, regardless of the value of *Λ*, external inputs are likely to contribute to the endemic level in a stationary environment and they are very likely to increase the re-infection rate as well.

From a strategic perspective, however, the most notable result of these analyses is that only three of the 10 villages, and a good argument can be made that it is three of the 20 original villages, appear to be able to sustain transmission without external inputs of cercariae or miracidia. This observation provides a compelling argument for the importance of connectivity, that is, a metapopulation perspective, in both endemic and re-emergent circumstances. A metapopulation perspective is supported by theoretical studies of Watt *et al*. [7]. In retrospect, some of the earliest investigations of our group using historical data collected in this area suggested the importance of connectivity although it was not appreciated at the time [18]. Additional support for the importance of hydrological connectivity, in particular, was provided by Xu *et al*. [19] who carried out large-scale simulation studies of schistosomiasis transmission in this region. Guararie & Seto [20] have shown theoretically that a network of hydrologically and socially connected villages can support endemic transmission without *any* of the individual villages on the network doing so if they were not connected. Hence, the results presented here, based on field data, lend support to the contention that connected networks of villages are the crucial unit for the purposes of both risk assessment and sustainable transmission control. While the crucial scale of this connectivity remains unclear, if correct, this interpretation suggests the need for the analogue of the air pollution control district, common in the USA, which is based on the characteristics of the airshed and of the source characteristics and distribution within it. These districts often cut across local political jurisdictions as would be necessary in the present case.

A second strategic point, also related to connectivity, arises from the observation that the lack of direct data from the field on C_E_(*t*), M_E_(*t*) and the two spatial parameters are the major limitation in assessing the sources driving transmission in a single village. A major step forward in addressing this need would be a sensitive, cheap and robust water monitoring procedure for the measurement of the parasite concentration in surface waters. For example, measuring total parasite concentrations in influent irrigation waters would allow estimation of the sum of C_E_(*t*) and M_E_(*t*). Similar measurements at various locations within the village would allow estimation of *γ* and *ξ* although the usefulness of both sets of measurements would be greatly enhanced by the development of advanced methods to distinguish cercariae from miracidia. Polymerase chain reaction (PCR) methods have been published for detection of the parasite [21,22] and preliminary work has been carried out in investigating field sampling methods with detection using qPCR methods [23]. These preliminary results hold the promise of circumventing the longstanding difficulty of cercarial monitoring of water [24].

Further motivation for the development of environmental sampling procedures for the detection of the free-swimming forms of the parasite is offered by circumstances now occurring in China. The extensive recent use of praziquantel and the molluscicide niclosamide has suppressed transmission levels difficult to detect by continued reliance on acute schistosomiasis case reports and episodic surveys for *S. japonicum*-infected humans and/or snails. For example, in 28 villages which had previously attained control status and were not known to currently support transmission, thorough surveys showed that human prevalence ranged from 0 to 27.8 per cent. Moreover, only one of 7515 snails collected in these villages was found to be infected despite extensive surveys at the time, these human infections were diagnosed [1]. Precedence for the importance of environmental surveillance has also been reported and shown to be effective for other waterborne pathogens, notably in the case of polio virus also in circumstances, as with schistosomiasis in Sichuan, where human case reports had previously provided the first warning of renewed transmission [25].
